# Perception of Dietary Influences on Renal Stone Formation Among the General Population

**DOI:** 10.7759/cureus.26024

**Published:** 2022-06-16

**Authors:** Ahmad Bashir, Sahar K Zuberi, Bazil Musharraf, Hasan Khan, M Hammad Ather

**Affiliations:** 1 Urology, Aga Khan University Hospital, Karachi, PAK; 2 Basic Sciences, Aga Khan University Hospital, Karachi, PAK; 3 Surgery, Aga Khan University Hospital, Karachi, PAK; 4 Urology, Department of Surgery, Aga Khan University, Karachi, PAK

**Keywords:** nutrition, questionnaire, prevention, diet, urolithiasis

## Abstract

Introduction

Urolithiasis is a common disorder worldwide with an increasing prevalence and high recurrence rate. This makes preventive measures like dietary modification an essential part of patient care. This study focuses on gauging the perception of dietary habits favoring kidney stone formation.

Materials and methods

A cross-sectional questionnaire-based study was conducted at Aga Khan University, Karachi, Pakistan. For nine food items and 14 beverages, respondents chose one of four options with regards to their relationship with stone formation, i.e. “increasing”, “decreasing”, “no effect”, and “do not know”. Responses were matched against evidence from the literature to generate correct and incorrect responses, thereby gauging perception for individual items.

Results

Seven hundred and three participants including 69 (9.6%) with a prior history of kidney stones, were recruited for the study. Participants with a personal history of kidney stone disease were older (odds ratio {OR}: 1.042 CI 1.020-1.064) with a significantly higher family history of stones (OR: 2.151 CI: 1.472-3.144). The majority were managed medically (87%) but never received dietary counseling (57%). Water, soft drinks, and tomatoes were the only three items out of 23 that were correctly identified by >50% of the participants with regards to their effect on stone formation. Responses did not differ significantly between those with stone disease and those without.

Conclusion

There is a lack of awareness among the general population, including individuals with a prior history of kidney stones regarding dietary prevention of kidney stone disease. This demonstrates a lack of existing dietary counseling thus necessitating the need for incorporating it at a mass level.

## Introduction

Urolithiasis is a common health disorder worldwide with a lifetime risk of stone formation as high as 10- 12% in males and 6-8% in females [1]. The prevalence of the stone disease is on the rise over the past two decades with an increasing burden of disease across gender, age, and race [2]. Existing evidence suggests that the prevalence of kidney stone disease in Pakistan is approximately 16% [3]. The prevalence of urolithiasis by age groups is as high as 31.5% in 40-49 year-olds, 29.6% in 30-39 year-olds, and 12.2% in 20-29 year-olds [4].

Renal stones and their management present as a major burden on health care resources and, when neglected, can go on to have detrimental effects on renal function. Different risk factors include poor dietary habits, inadequate water intake, and comorbidities like diabetes, hypertension, dyslipidemia, obesity, coronary artery disease, and depression [5-8]. If preventive strategies are not employed, the 5-year rate of recurrence of kidney stones is 50% [9]. This high risk of recurrence substantiates the significance of dietary counseling in patients with kidney stones. With little or incomplete resources regarding dietary habits to curtail stone formation available to the public, it is essential that any patient who presents to a hospital should receive dietary counseling [10].

To the best of our knowledge, only one study has previously aimed to gauge the perception of the population regarding the influence of commonly used dietary items. The afore-mentioned study was conducted in the United States and so understandably will differ significantly owing to the difference in dietary habits in this part of the world [11].

The American Urological Association guidelines identify several food and beverage items that are categorized as “stone favorable” or “stone unfavorable” with regards to their role in stone formation [12]. An understanding of patients' perceptions can not only validate the current practices but can also lead to the identification of areas requiring special attention. Ultimately, it will result in the reduction of the health care burden and will also lower the rates of stone formation and recurrence in the general population.

## Materials and methods

An analytical cross-sectional survey was conducted within the confines of our tertiary care hospital, with the cohort comprising of attendants waiting in outpatient consulting clinics. The Institutional Ethics Review Committee (ERC) issued approval for this study with approval number ERC: 2020-3566-11131. The study was given approval for a period of one year with effect from 01-Jul-2020. The duration of data collection to attain desired sample size was 12 weeks from the approval date.

The sample size was calculated on open Epi software version 3.01 (Estimation Programs Interface Suite™ for Microsoft® Windows, United States Environmental Protection Agency, Washington, DC). The minimum sample size that was required was 703 participants with an inflation of 6% for the non-response rate. The anticipated proportion of patients who do not have knowledge of dietary influence on renal stone formation was set at 50% with a precision of 5%, confidence level of 99%, and a design effect of 1. The eligibility criteria included participants above 18 years of age who would give informed consent, and have the ability to read and understand either the English or Urdu language. Data were collected by two of the authors, by handing a standard survey questionnaire to attendants of patients in the waiting area of consultant clinics while being present to answer any queries. No individual other than the authors had access to the data. The survey included basic demographic details, personal and family history of stone disease, and a list of 14 food and nine beverage items. All items were listed in tabular form, with the participants having to select one of “stone favorable”, “stone unfavorable” and “stone neutral” for each item. Additionally, a fourth column titled “Don’t know” could also be marked.

Data analysis

Respondents were divided into two groups, stone formers, and non-stone formers, with further stratification based on age, gender, level of education, and past history. Data was analyzed on SPSS software version 23.0 (IBM Corp., Armonk, NY). The qualitative variables were reported as frequencies and percentages. The relationship of these variables, as well as the correct identification of items that increase or decrease stone favorability with the status of stone formation, was assessed by Chi-square/Fisher’s exact test. Univariate and multivariate analysis was done for age and family history of kidney stone disease to calculate crude and adjusted odd’s ratio with 95% CI. Quantitative variables were described as mean ± standard deviation. P-value of <0.05 was considered as significant throughout the study.

## Results

The age of participants ranged from 18 to 80 years with a mean age of 34.6 ± 10.8 years. Three hundred and eighty-seven (55%) of the participants were males and 316 (45%) were females. Most of them were residents of Sindh (639, 89.8%) with graduate-level education (486, 69.1%). Two hundred and seventy (38.4%) respondents had a family history and (69, 9.8%) had a personal history of kidney stone disease. One respondent left the question about residence and one left family history unanswered. Both stone formers and non-stone formers displayed male preponderance with graduate-level education. The association of age, gender, level of education, residence, and family history of kidney stone disease to an individual’s personal history of kidney stones is displayed in Table 1. Stone formers were more likely to have a family history of kidney stone disease (odd ratio {OR} 2.1) and be above 30 years of age. With every one-year increase in age, the odds of having a history of kidney stones increased by 4.2%.

**Table 1:** Association between demographic parameters and personal history of kidney stones *significant (p-value<0.05). Chi-squares/Fisher exact tests were used. Univariate regression analysis was done for odds ratio (OR) at a 95% confidence interval (95% CI).

	History of Kidney Stone		No history of kidney stone		p-value	OR (95% CI)
	N (N=69)	%	N (N=634)	%		
Age (years)						
18-29	14	20.3	244	38.5	<0.001*	1.042 (1.020 – .064)*
30-49	37	53.6	322	50.8		
>50	18	26.1	68	10.7		
Mean ± sd	39.5 ± 11.5		34.1 ± 10.6			
Gender						
Male	43	62.3	344	54.3	0.201	1.394 (0.86-2.325)
Female	26	37.7	290	45.7		
Education Level						
Up to primary school	4	5.8	22	3.5	0.468	
Up to high school	20	29	171	27		
Graduate	45	65.2	441	69.5		
Residence						
Sindh	58	84.1	573	90.5	0.313	
Punjab	3	4.3	13	1.9		
Baluchistan	5	7.2	30	4.7		
Khyber Pakhtunkhwa	2	2.9	5	0.8		
Islamabad	0	0	3	0.5		
Not from Pakistan	1	1.4	10	1.6		
Family History of Renal Stone						
Yes	38	55.1	232	36.7	0.004*	2.151 (1.472-3.144)*
No	31	44.9	401	63.3		

**Figure 1:**
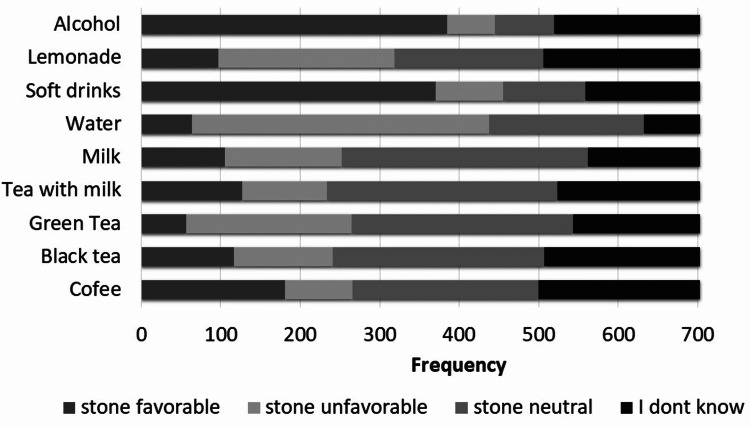
Perception of favorability of kidney stone formation of different beverages

**Figure 2:**
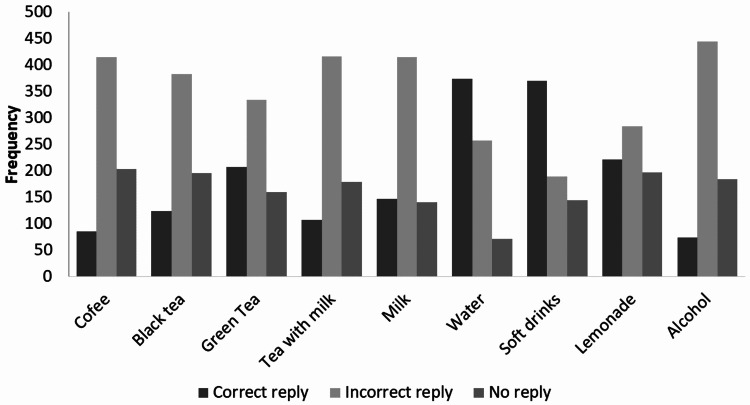
Frequency of correct and incorrect replies regarding stone favorability of beverages

Most participants believed that soft drinks (52.6%) and alcohol (54.8%) were stone favorable; water (53.2%) was stone unfavorable while coffee (33.3%), black tea (37.8%), green tea (39.5%), tea with milk (41.3%) and milk (44.1%) were stone neutral (Figure 1). Soft drinks (52.6%) and water (53.2%) were the only items correctly identified by more than 50% of participants (Figure 2).

**Figure 3:**
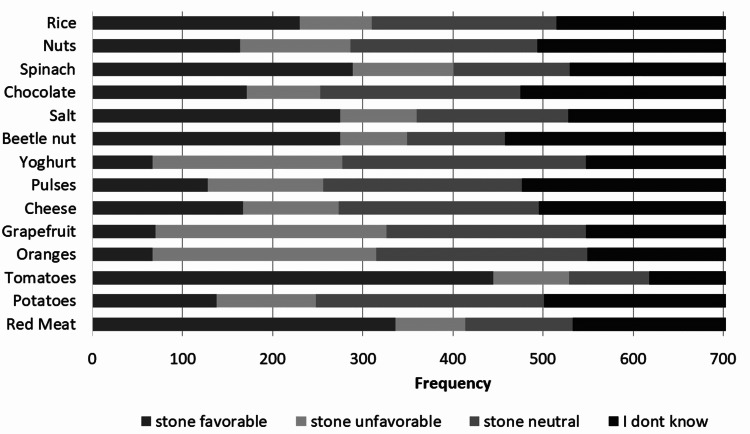
Perception of favorability of kidney stone formation of different food items

**Figure 4:**
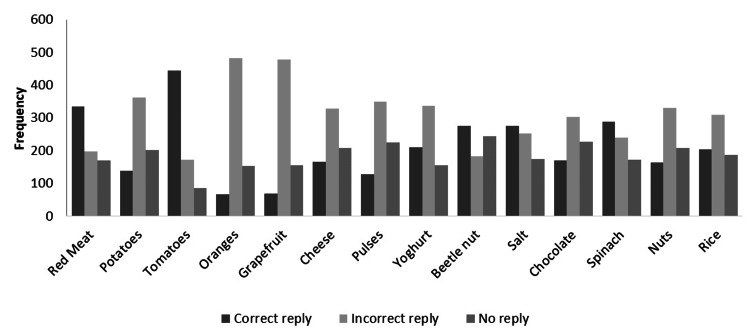
Frequency of correct and incorrect replies regarding stone favorability of food item

Most participants also believed that red meat (47.8%), tomatoes (63.3%), beetle nut (39.1%), salt (39.1%), spinach (41.1%), and rice (32.7%) were stone favorable; oranges (35.3%) and grapefruit (36.4%) were stone unfavorable while potatoes (36%), cheese (31.6%) and yogurt (38.5%) were stone neutral (Figure 3). However, only red meat (47.8%), tomatoes (63.3%), beetle nut (39.1%), salt (39.1%), and spinach 289 (41.1%) were the items correctly identified by a significant proportion of people (Figure 4).

Out of the 69 stone formers, the nature of treatment sought by individuals with kidney stone disease included medical management (75.4%) and surgical intervention (11.6%) but approximately half of them (43%) denied having received dietary counseling. Their responses did not differ from non-stone formers for all items except potatoes, spinach, and beetle nut (Table 2).

**Table 2:** Frequency and odds ratio of correctly identifying the relationship of food items with likelihood of kidney stone formation in individuals with and without kidney stone disease *=significant (p-value<0.05). Univariate and multivariate regression analysis was done to calculate the odds ratio (OR) with a 95% confidence interval (95%CI). OR was adjusted for age and family history of kidney stone disease.

	History of Kidney Stone (N=69)		No history of kidney stone (N=634)		OR (95% CI)		Adjusted OR (95% CI)
	Correct Reply N (%)	Incorrect Reply N (%)	Correct Reply N (%)	Incorrect Reply N (%)			
Beverages							
Coffee	7 (10.1)	62 (89.9)	78 (12.3)	556 (87.7)	0.805 (0.356-1.821)		
Black Tea	16 (23.2)	53 (76.8)	108 (17)	526 (83)	1.470 (0.81-2.669)		
Green Tea	20 (29)	49 (71)	188 (29.7)	446 (70.3)	0.968 (0.560-1.674)		
Tea with Milk	7 (10.1)	62 (89.9)	100 (15.8)	534 (84.2)	0.603 (0.268-1.355)		
Water	38 (55.1)	31 (44.9)	336 (53)	298 (47)	1.087 (0.660-1.791)		
Lemonade	19 (27.5)	50 (72.5)	203 (32)	431 (68)	0.807 (0.486-1.565)		
Milk	10 (14.5)	59 (85.5)	137 (21.6)	497 (78.4)	0.615 (0.464-1.404)		
Alcohol	4 (5.8)	65 (94.2)	70 (11)	564 (89)	0.496 (0.175-1.402)		
Soft drinks	41 (59.4)	28 (40.6)	329 (51.9)	305 (48.1)	1.357 (0.819-2.250)		
Food							
Red meat	37 (53.6)	32 (46.4)	299 (47.2)	335 (52.8)	1.295	(0.787-2.132)	
Potatoes	22 (31.9)	47 (68.1)	116 (18.3)	518 (81.7)	2.090	(1.212-3.604)*	2.041 (1.163-3.581)*
Tomatoes	42 (60.9)	27 (39.1)	403 (63.6)	231 (36.4)	0.892	(0.536-1.485)	
Orange	4 (5.8)	65 (94.2)	63 (9.9)	571 (90.1)	0.558	(0.197-1.582)	
Grapefruit	8 (11.6)	61 (88.4)	62 (9.8)	572 (90.2)	1.210	(0.553-2.645)	
Cheese	21 (30.4)	48 (69.6)	146 (23)	488 (77)	1.462	(0.848-2.522)	
Pulses	7 (10.1)	62 (89.9)	121 (19.1)	513 (80.9)	0.479	(0.214-1.072)	
Yoghurt	19 (27.5)	50 (72.5)	191 (30.1)	443 (69.9)	0.881	(0.506-1.535)	
Beetle nut	19 (27.5)	50 (72.5)	256 (40.4)	378 (59.6)	0.561	(0.323-0.974)*	0.535 (0.305-0.940)*
Salt	31 (44.9)	38 (55.1)	244 (38.5)	390 (61.5)	1.304	(0.790-2.151)	
Chocolate	17 (24.6)	52 (75.4)	154 (24.3)	480 (75.7)	1.019	(0.572-1.814)	
Spinach	21 (30.4)	48 (69.6)	268 (42.3)	366 (57.7)	0.597	(0.349-1.022)	0.523 (0.301-0.909)*
Nuts	17 (24.6)	52 (75.4)	147 (23.2)	487 (76.8)	1.083	(0.608-1.930)	
Rice	15 (21.7)	54 (78.3)	190 (30)	444 (70)	0.649	(0.357-1.179)	

## Discussion

American Urological Association has identified dietary counseling as a keystone for the prevention of kidney stone disease [12]. Without preventive measures, the 5-year rate of recurrence is approximately 50%, and a staggering 75% in 20 years [9]. This makes it essential to employ all possible preventive measures to avoid recurrent episodes of kidney stone disease.

Since dietary counseling is an essential element in the prevention of kidney stones, the assessment of baseline awareness of the population regarding dietary habits that increase or decrease the likelihood of kidney stone formation is imperative. Unfortunately, no such study has been carried out on the Pakistani population to date. Hence, this article focuses on gauging the perception of the general population regarding the stone favorability of several commonly used food items and beverages.

The demographic parameters including age and gender are consistent with previously published data which showed that kidney stones are more prevalent in Pakistani individuals above 30 years of age with a preponderance of the male gender [4,13,14]. Similar to previously published data, individuals with a positive family history of kidney stones were found to be at a higher risk of developing kidney stones, odd’s ratio of 2.1 in our study [14-17].

The prevalence of kidney stone disease was found to be 9.8% which is lower than the previously reported 16.6% [18] which could be due to the majority of the cohort belonging to the province of Sindh. 87% of those diagnosed with kidney stone disease were managed medically, and only 57% received dietary counseling highlighting the lack of emphasis on the matter. Moussa et al. reported that merely 32% of the patients were counseled regarding kidney stone prevention in the Emergency Department (ED) setting in Saudi Arabia [19]. The low dietary counseling could be explained by the loss of follow-up from the emergency departments with no dietary counseling by ED physicians. This demonstrates that dietary counseling is essential at all levels of healthcare.

This study highlights the lack of awareness of the population regarding dietary habits that favor kidney stone formation. Interestingly, even individuals with a personal history of kidney stones could not correctly identify most stone favorable food and beverage items. This differs from a study published by Alghamdi et al. and Marsh et al. where individuals with a history of kidney stones demonstrated better knowledge of dietary habits that favor kidney stone formation [11,20]. 

The only food and beverages correctly identified by more than 50% of the participants were water, soft drinks, and tomatoes. The notion of vilified food items presented by Marsh et al. applies to this population stratum as well. Coffee, tea, and alcohol are considered unhealthy and have thus been marked as stone favorable by most respondents when they in reality have a protective effect on kidney stone formation. Interestingly, in contrast to the findings in Marsh et al. study, despite being considered healthy, spinach was correctly identified as stone favorable by most [11]. Strangely so, fewer kidney stone formers correctly identified spinach and beetle nut as stone favorable as compared to the general population. However, potatoes were correctly identified by more kidney stone formers. These findings of an erratic pattern of knowledge shed light on the lack of awareness of the population regarding dietary habits that promote kidney stone formation irrespective of stone status.

The strengths of this study include highlighting the lack of awareness of the general population of Pakistan regarding the influence of dietary habits on kidney stone formation. It also focuses attention on the lack of dietary counseling for kidney stone patients on the part of clinicians. These findings could aid in the development of strategies to ascertain that the masses are aware of the preventive measures to avoid stone formation. This could be done by designing dietary sheets with the help of nutritionists and making them available in all departments these patients present to, including urologists, nephrologists, emergency medicine physicians, etc.

There are various limitations of this research which include its observational study design, non-validated questionnaire, and convenience sampling. A non-validated questionnaire was used so all cultural food items could be included. Moreover, this is a single-center study with the majority of participants belonging to a single city of a single province, and therefore the study results can be generalized to the population presenting to private tertiary care hospitals in Pakistan only. Since it was conducted in a private hospital, education and socioeconomic status could be a potential confounder, as patient strata in private hospitals are more likely to be educated. Less educated people might be even less aware of any preventive dietary habits.

Unlike most hospitals in Pakistan, this center has certified nutritionists to whom physicians can refer their patients. Therefore, participants being present in this institute are more likely to have received dietary counseling.

## Conclusions

There is a lack of awareness in the population of Pakistan, irrespective of whether they have a history of kidney stones or not, regarding dietary modifications that can prevent kidney stone formation. Thus highlighting the lack of dietary counselling for patients with kidney stone disease. These findings also suggest that the development of a standardized dietary sheet will prove beneficial.

## Appendices

**Table 3:** Favorability of food items mentioned in the questionnaire in promoting kidney stone formation Reference no. [12]

Stone favorability	Food items
Stone favorable	soft drinks, red meat, potatoes, tomatoes, orange, grapefruit, cheese, pulses, beetle nut, salt, chocolate, spinach, nuts
Stone neutral	alcohol, rice
Stone unfavorable	coffee, black tea, green tea, tea with milk, water, lemonade, milk, yoghurt
